# Chance diagnosis of mixed gonadal dysgenesis in an adult case of malignant gonadal germ cell tumor: a case report

**DOI:** 10.1186/s13256-021-02758-w

**Published:** 2021-03-28

**Authors:** Yoshiko Kurose, Tomonori Nagai, Kousuke Shigematsu, Takahiro Uotani, Taichi Akahori, Yasushi Takai, Hiroyuki Seki

**Affiliations:** Saitama Medical Center, Saitama Medical University, 1981 Kamoda, Kawagoe, Saitama 350-8550 Japan

**Keywords:** Mixed gonadal dysgenesis, Primary amenorrhea, Gonadal tumor, 45,X/46,XY mosaicism, Disorders of sex development, Case report

## Abstract

**Background:**

Mixed gonadal dysgenesis (MGD) is a subtype of the disorders of sex development (DSD) associated with sex chromosome abnormalities characterized by abnormal external genitalia, short stature, and primary amenorrhea. This disease is generally diagnosed from the neonatal stage to early childhood, and by puberty at the latest. Cases that are phenotypically female or those with ambiguous genitalia experience a high risk of gonadal tumor formation. As tumor risk is known to increase with age, prophylactic bilateral gonadectomy is recommended following early diagnosis.

**Case presentation:**

Here we report a case of an adult Japanese woman diagnosed with MGD during treatment for a giant pelvic tumor. The patient initially visited a gynecology clinic during puberty for primary amenorrhea, at which time an abnormality was found with the external genitalia. However, a diagnosis of MGD was not made at this time, resulting in the development of a malignant gonadal germ cell tumor in adulthood.

**Conclusions:**

For early diagnosis of MGD and the prevention of gonadal tumor formation, it is essential that gynecologists fully understand MGD and other DSD.

## Background

Mixed gonadal dysgenesis (MGD) is a subtype of the disorders of sex development (DSD) associated with sex chromosome abnormalities. The incidence of this rare disease is less than 1 per 15,000 births [1]. The most common form of the disease is a karyotype displaying 45,X/46,XY mosaicism, which accounts for 35% of patients [2]. Patients have unilateral dysgenetic testis and contralateral streak gonads, as well as abnormal differentiation of related internal and external sex organs. Phenotypes range from normal male form, to ambiguous genitalia, to forms that closely resemble a normal female.

MGD is commonly diagnosed from as early as the neonatal to infant stage based on abnormal external genitalia, or is otherwise diagnosed as late as puberty based on reduced stature and primary amenorrhea.

Among DSD, MGD carries a high risk of gonadal tumor development. Patients who are phenotypically female or possess ambiguous genitalia are at increased risk compared to male-type patients. The risk of gonadal tumor onset rises with age, as does the risk that malignant germ cell tumors such as dysgerminomas and seminomas will develop. For this reason, it is recommended that phenotypic females or those with ambiguous genitalia undergo prophylactic bilateral gonadectomy at an early stage following a diagnosis of MGD [1, 3].

We experienced a rare case where a phenotypically female MGD patient experienced primary amenorrhea yet reached adulthood without undergoing more careful examination. This resulted in a chance diagnosis of MGD during treatment for a giant pelvic tumor at the age of 25 years. MGD is a disease often diagnosed by a pediatrician, but when a general gynecologist encounters a female patient with primary amenorrhea, MGD must be considered as a differential diagnosis, as this disease requires the correct diagnosis and prophylactic bilateral gonadectomy before a gonadal tumor develops. Here we describe a rare case in which a patient reached adulthood before being diagnosed with MGD, which was discovered by chance during medical treatment of a malignant gonadal germ cell tumor.

## Case presentation

This case involves an unmarried 25-year-old phenotypic Japanese female, 154.1 cm in height and 48.5 kg in weight, and without a notable medical, family, or psychosocial history. She did not consume alcohol, tobacco, or any drugs. The patient was examined at the age of 18 at a gynecology clinic for primary amenorrhea where, given the patient’s active involvement in competitive track and field, she was diagnosed with athletic amenorrhea without further tests being conducted. Afterwards, the patient underwent no treatment and did not experience menstruation. Moreover, the patient had not engaged in sexual intercourse up to this point.

**Fig. 1:**
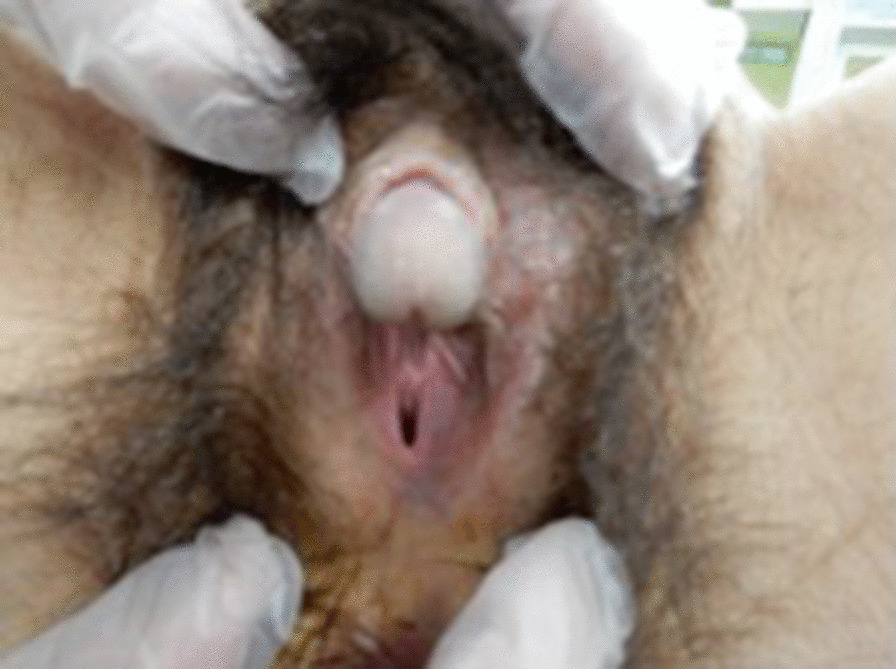
External genital abnormalities: clitoral hypertrophy and narrowing of the vaginal opening

**Fig. 2:**
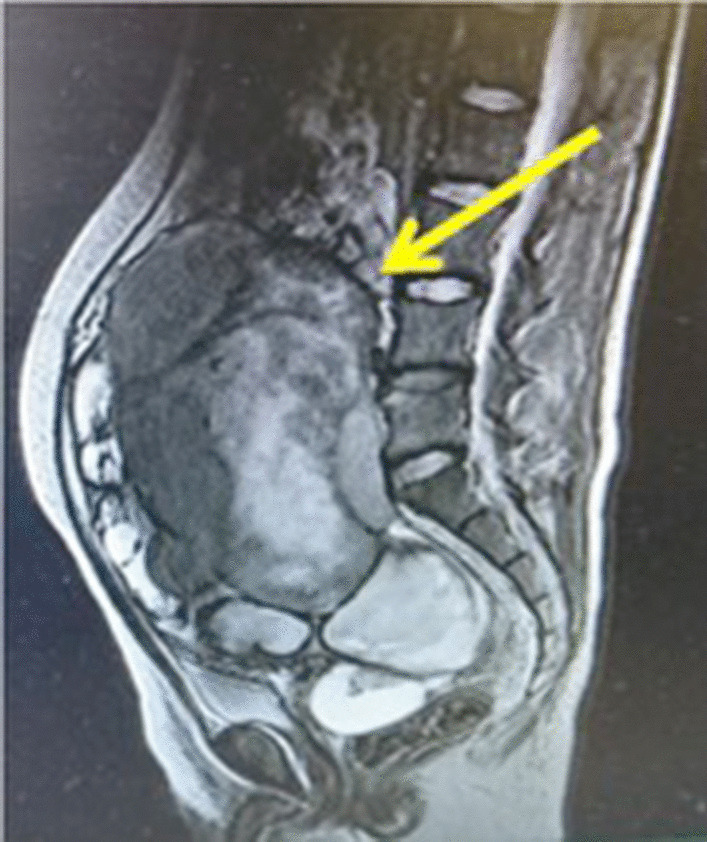
Magnetic resonance imaging findings (T2-weighted image): pelvic tumor with a mixture of solid and cystic components (arrow)

Upon examination by a local physician with the main complaint of abdominal distention, the patient was referred to our hospital with a suspected ovarian tumor, as a giant 17 cm tumor was found in the pelvis. The inspection of the external genitalia revealed clitoral hypertrophy and narrowing of the vaginal opening (Fig. 1). Pelvic magnetic resonance imaging (MRI) revealed a tumorous lesion in the pelvis measuring 218 × 115 × 178 mm, containing a mixture of cystic and solid components. The uterus could not be found. The cystic components contained blood, and the solid portion had a contrast effect (Fig. 2). Examination by ^18^F-fluorodeoxyglucose (FDG)-positron emission tomography/computed tomography revealed FDG accumulation in the tumor and in enlarged abdominal para-aortic lymph nodes. Blood tests revealed the following levels: white blood cells (WBC): 10.7/μL × 10^3^; hemoglobin: 10.1 g/dL; platelets: 412/μL × 10^4^; C-reactive protein (CRP): 18.1 mg/dL; total protein: 7.7 g/dL; albumin: 3.8 g/dL; urea nitrogen: 8 mg/dL; creatinine: 0.53 mg/dL; total bilirubin: 0.6 mg/dL; aspartate aminotransferase (AST): 30 IU/L; alanine aminotransferase (ALT): 8 IU/L; follicle-stimulating hormone (FSH): 52.4 mU/mL; luteinizing hormone (LH): 34.4 mU/mL; estradiol (E2): < 10 pg/mL; progesterone (P4): 0.66 ng/mL; and testosterone: 0.25 ng/mL. Serum alpha-fetoprotein (2.4 ng/mL) was within the normal range, but serum beta-human chorionic gonadotropin (5.86 ng/mL), lactate dehydrogenase (4574 µg/L), and CA125 (165 µg/mL) were elevated. Urinalysis was normal. As DSD was suspected because of the appearance of the pelvic tumor, chromosome testing was performed. The karyotype was found to exhibit 45,X/46,XY mosaicism, and the patient was diagnosed with MGD. Since the pelvic tumor was considered to be a gonadal tumor, a resulting complication of MGD, an exploratory laparotomy was performed in order to arrive at a definitive pathologic diagnosis of the tumor.

**Fig. 3:**
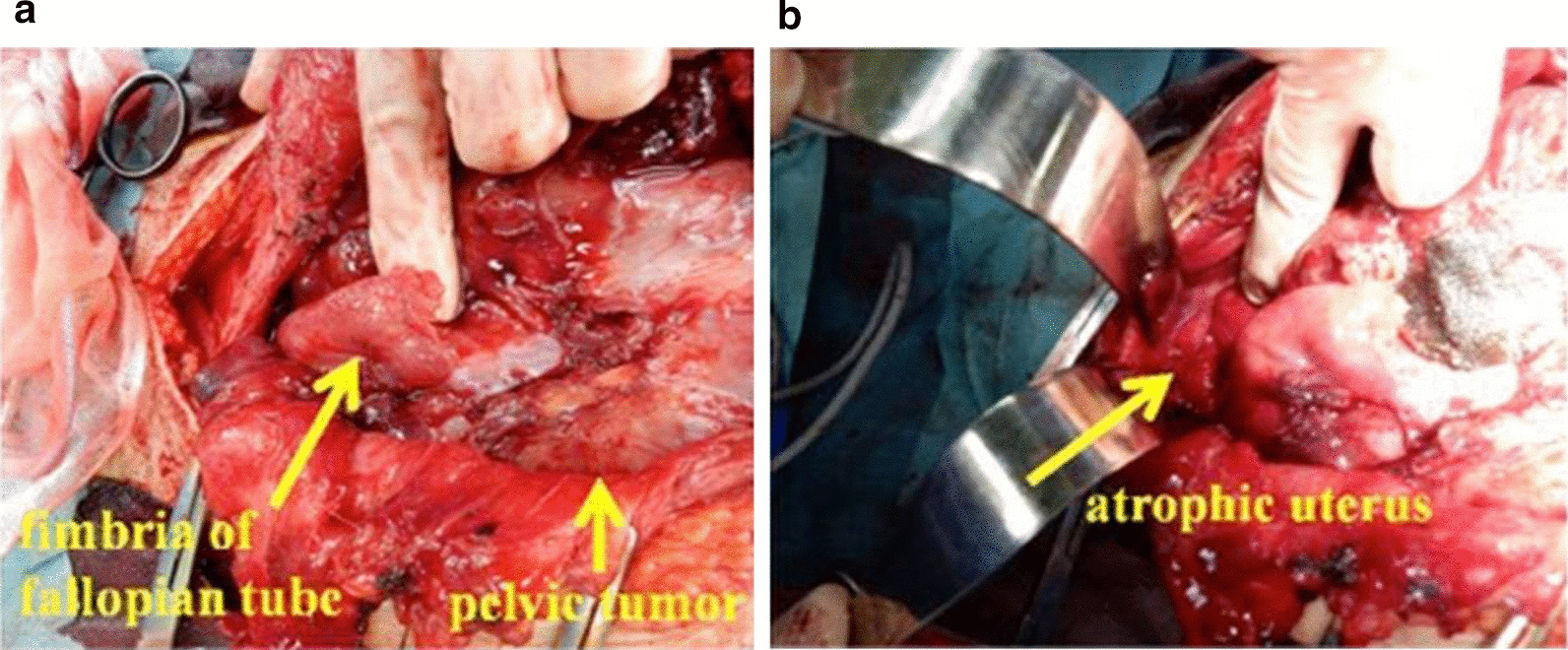
**a**, **b** Intraoperative findings (initial surgery): pelvic tumor, fallopian tube infiltration, and atrophy of the uterus

**Fig. 4:**
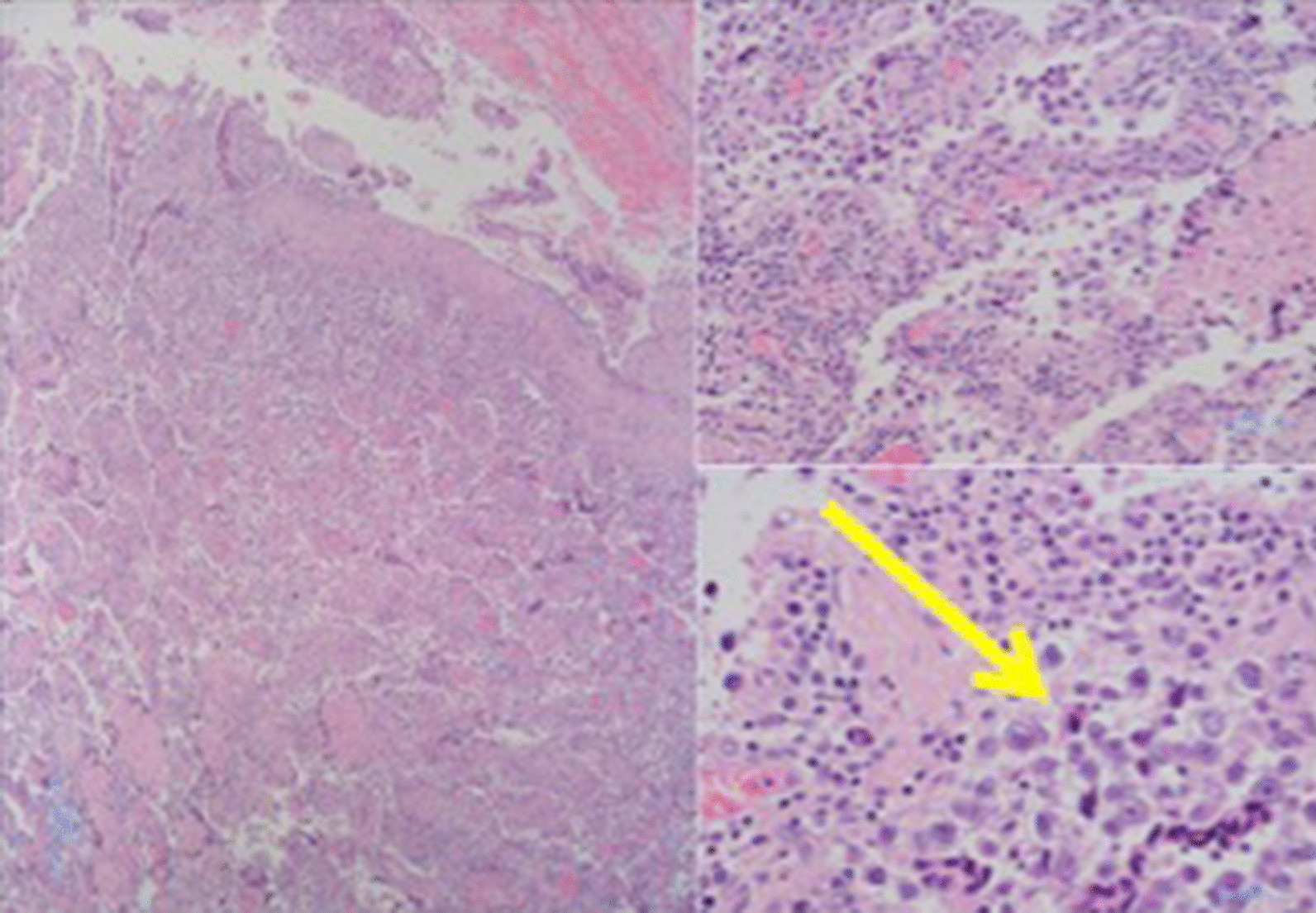
Tumor pathology findings (initial surgery): recognized "two-cell pattern" with tumor cells and lymphocyte infiltration (arrow)

A physical examination revealed a patient who looked unwell, with blood pressure of 119/77 mmHg, heart rate of 117 beats per minute, and body temperature of 38.7 °C. Neurological examination was unremarkable and did not offer any substantial information. Surgical findings revealed marked adhesions between the tumor and the surrounding organs, making excision difficult. Therefore, only a biopsy of the tumor was performed (Fig. 3a, b). The final pathological diagnosis was “seminoma” (Fig. 4). Consequently, the patient was diagnosed with clinical stage IIB testicular cancer (seminoma) with MGD.

**Fig. 5:**
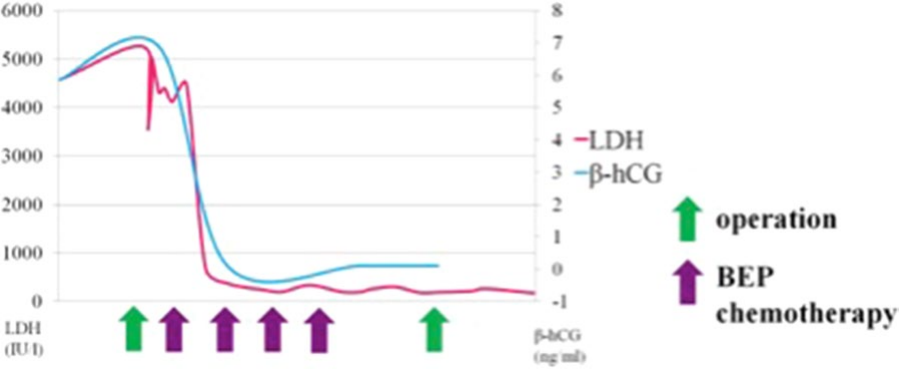
Clinical course table: elevated lactate dehydrogenase and beta-human chorionic gonadotropin rapidly declined after the start of bleomycin, etoposide, and cisplatin (BEP) therapy

**Fig. 6:**
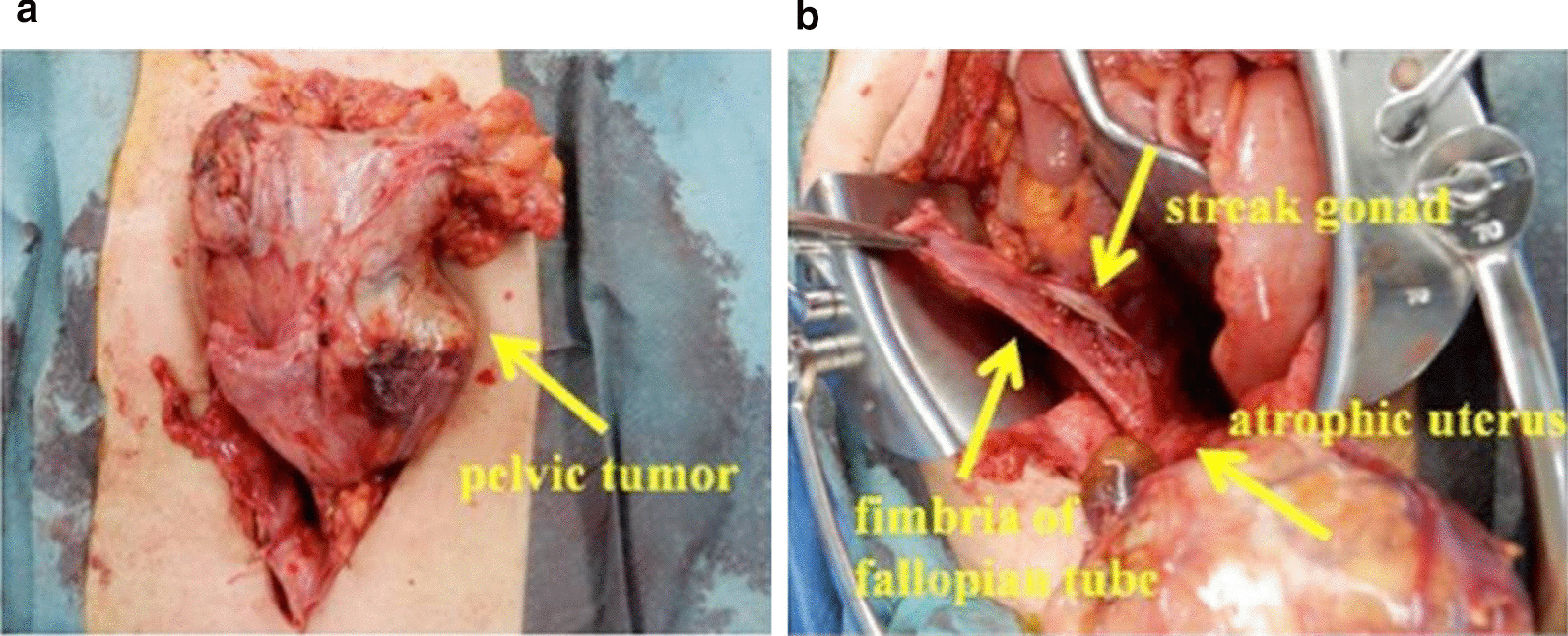
**a**, **b** Intraoperative findings (second surgery): pelvic tumor reduced by chemotherapy, and atrophy of the uterus, fallopian tubes, and streak gonad

**Fig. 7:**
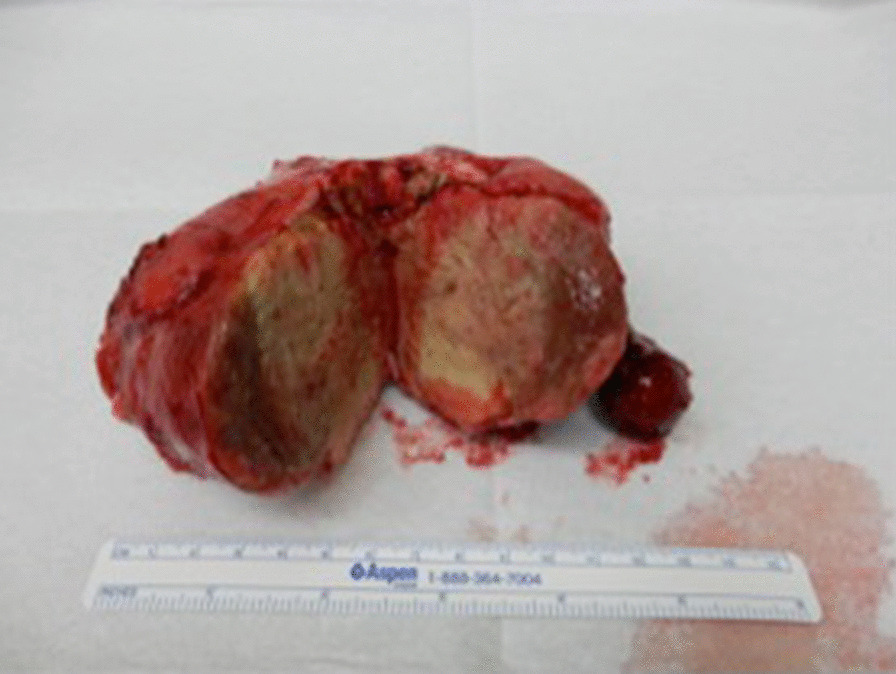
Macroscopic image of excised specimen (second surgery): tumor exhibiting uniform yellowish-white color and conspicuous necrosis inside the tumor

Postoperative systemic chemotherapy was started promptly, consisting of bleomycin, etoposide, and cisplatin (BEP). At the time of the fourth course of BEP, tumor markers had normalized (Fig. 5). Marked tumor reduction was confirmed with imaging, and subsequently the patient underwent interval debulking surgery. Surgery involved bilateral gonadectomy and resection of disseminated peritoneal lesions (Figs. 6a, b, 7). Pathological examination of the excised specimens was consistent with necrosis of the seminoma due to the effect of chemotherapy. No viable tumor remains were found. No further chemotherapy was administered, and the initial course of treatment was completed. We notified the patient regarding MGD, and she decided her future sex as female. Therefore, we proposed some surgical options for clitoromegaly or vaginal stenosis. However, she did not wish to pursue those at that time.

Currently, 4 years and 11 months after the initial treatment, there has been no recurrence of the tumor. In addition, the patient has been on postoperative hormone replacement therapy in the form of an estradiol/norethindrone acetate patch and psychotherapy continuously.

## Discussion

Of note in this case is the delay in diagnosis despite the presence of clinical features such as external genital abnormality and primary amenorrhea. Indeed, diagnosis was delayed until the patient was 25 years old and was only discovered by chance during treatment for a malignant gonadal germ cell tumor.

MGD is a disease that may be diagnosed based on short stature, abnormal external genitalia, or primary amenorrhea. Moreover, it is normally a disease that can be diagnosed by puberty at the latest. Berberoglu *et al*. studied 12 cases of 45X/46,XY GD, and found that the average age of diagnosis was 5.14 years. They further reported that 50% of cases involved short stature [4]. A study by Huang *et al*. of 19 MGD cases reported that the median age at diagnosis was 11 years 5 months (age range: 1 month–15 years 9 months), and indicated short stature (74%), abnormal external genitalia (37%), and primary amenorrhea (5%) as the three triggers for diagnosis. Three of the 19 cases (16%) were ultimately diagnosed with gonadoblastoma through preventive gonadectomy, but there were no cases that were clinically diagnosed as a result of a pelvic tumor [5].

One of the key clinical issues associated with DSD is gonadal tumor onset, and MGD is reported to be the DSD with the highest risk of gonadal tumor development [6]. This is thought to be due to the presence of Y chromosome sequences in GD patients, which increases the risk of gonadal tumor formation [7]. Studies indicate that the risk of tumor onset is 3-4% at age 10, increasing with age to 10–20% at age 15, and to as high as 46% at age 40 [3]. The most common gonadal tumors are benign gonadoblastomas, but malignant germ cell tumors such as dysgerminomas and seminomas also frequently occur [8]. The risk of developing gonadal tumors is higher among phenotypic female and ambiguous genitalia MGD cases than in male-type cases. Moreover, while resection of streak gonads is recommended at an early stage in male-type cases, while sparing the contralateral dysgenetic gonad and carefully monitoring the patient, early prophylactic bilateral gonadectomy in infancy is recommended for female or ambiguous genitalia cases [1, 3].

In the case described here, abnormality of the external genitalia (clitoral hypertrophy and narrowing of the vaginal opening) were indeed present. If the initial gynecologist had been better acquainted with DSD so as to recognize its signs when the patient was examined at 18 years of age, further testing could have led to a correct diagnosis. However, there is a general lack of understanding among gynecologists with respect to DSD. In this case, that lack of awareness resulted in a malignant gonadal germ cell tumor in adulthood, at which time MGD was finally diagnosed. The MGD cases that a gynecologist is likely to encounter are phenotypic females with an increased risk of gonadal tumor formation. In order to prevent the formation of malignant gonadal germ cell tumors, diagnosis as early as possible followed by prophylactic bilateral gonadectomy is necessary. Therefore, it is essential for gynecologists to understand the concept of DSD, as they will have many opportunities to treat adolescent women for primary amenorrhea.

## Conclusions

MGD is generally a disease that can be diagnosed by puberty at the latest. The delayed diagnosis in this case resulted in development of a malignant gonadal germ cell tumor. Gynecologists must be sufficiently aware of the disease concept of DSD, including MGD, in order to diagnose MGD early and prevent the onset of gonadal tumor formation.

## Data Availability

All data generated or analyzed during this study are included in this published article.
